# Cell Elasticity Determines Macrophage Function

**DOI:** 10.1371/journal.pone.0041024

**Published:** 2012-09-18

**Authors:** Naimish R. Patel, Medhavi Bole, Cheng Chen, Charles C. Hardin, Alvin T. Kho, Justin Mih, Linhong Deng, James Butler, Daniel Tschumperlin, Jeffrey J. Fredberg, Ramaswamy Krishnan, Henry Koziel

**Affiliations:** 1 Division of Pulmonary, Critical Care, and Sleep Medicine, Department of Medicine, Beth Israel Deaconess Medical Center, Harvard Medical School, Boston, Massachusetts, United States of America; 2 Key Laboratory of Biorheological Science and Technology, Ministry of Education, Bioengineering College, Chongqing University, Chongqing, China; 3 Program in Molecular and Integrative Physiological Sciences, Department of Environmental Health, Harvard School of Public Health, Boston, Massachusetts, United States of America; 4 Division of Health Sciences and Technology, Harvard–Massachusetts Institute of Technology, Cambridge, Massachusetts, United States of America; 5 Children's Hospital Informatics Program, Boston, Massachusetts, United States of America; National Jewish Health and University of Colorado School of Medicine, United States of America

## Abstract

Macrophages serve to maintain organ homeostasis in response to challenges from injury, inflammation, malignancy, particulate exposure, or infection. Until now, receptor ligation has been understood as being the central mechanism that regulates macrophage function. Using macrophages of different origins and species, we report that macrophage elasticity is a major determinant of innate macrophage function. Macrophage elasticity is modulated not only by classical biologic activators such as LPS and IFN-γ, but to an equal extent by substrate rigidity and substrate stretch. Macrophage elasticity is dependent upon actin polymerization and small rhoGTPase activation, but functional effects of elasticity are not predicted by examination of gene expression profiles alone. Taken together, these data demonstrate an unanticipated role for cell elasticity as a common pathway by which mechanical and biologic factors determine macrophage function.

## Introduction

Macrophages, despite arising from a common monocyte-granulocyte lineage, perform a myriad of functions such as host defense from infection, control of inflammation, and repair of injury [1], [2], [3] in order to preserve organ homeostasis. This is well demonstrated in the lungs where alveolar macrophages are the first line of host defense and serve as critical activators of inflammation promoting recruitment of neutrophils and other immune cells during the early stages of pneumonia [4]. Later in the course of pneumonia, alveolar macrophages are critical for controlling inflammation, limiting collateral damage, and promoting resolution through phagocytosis of bacteria, apoptotic host cells, and debris [5], [6]. While lower respiratory tract infections comprise the single largest burden of disease worldwide as assessed by disability adjusted life years (DALYS) [7], there remains limited understanding of how dynamic regulation of macrophage function is achieved to preserve lung function. Regulation of macrophage function is presently understood mainly in the context of ligation events and downstream signaling [2], [3] and attempts at describing macrophage phenotype have largely focused on changes in gene expression [8]. In the lungs, where mechanics play a critical role in determining organ function [9], pneumonia is typically associated with large increases in local tissue rigidity and decreases in tissue stretch [10], [11]. It is also well recognized that biologic modulators such as bacterial pathogen associated molecular patterns (PAMPs) or cytokines increase both lung tissue rigidity [12], [13], and cell elasticity [14], [15], although the role of cell mechanics in determining macrophage function are unclear.

Here we show that changes in macrophage elasticity (elastic modulus) exert a dominant influence on macrophage function. Importantly, macrophage elasticity is dynamic, is mediated by actin polymerization and RhoGTPase activation, and is independently influenced to a similar extent by biological factors (such as IFN-γ and LPS), substrate rigidity, and substrate stretch. These findings support the concept that macrophage innate function is determined not only by soluble biological factors, but also by organ or tissue physical factors through the unexpected and previously underappreciated role of macrophage elasticity.

## Results

### LPS and IFN-γ increase phagocytosis through effects on cell elasticity

**Figure 1:**
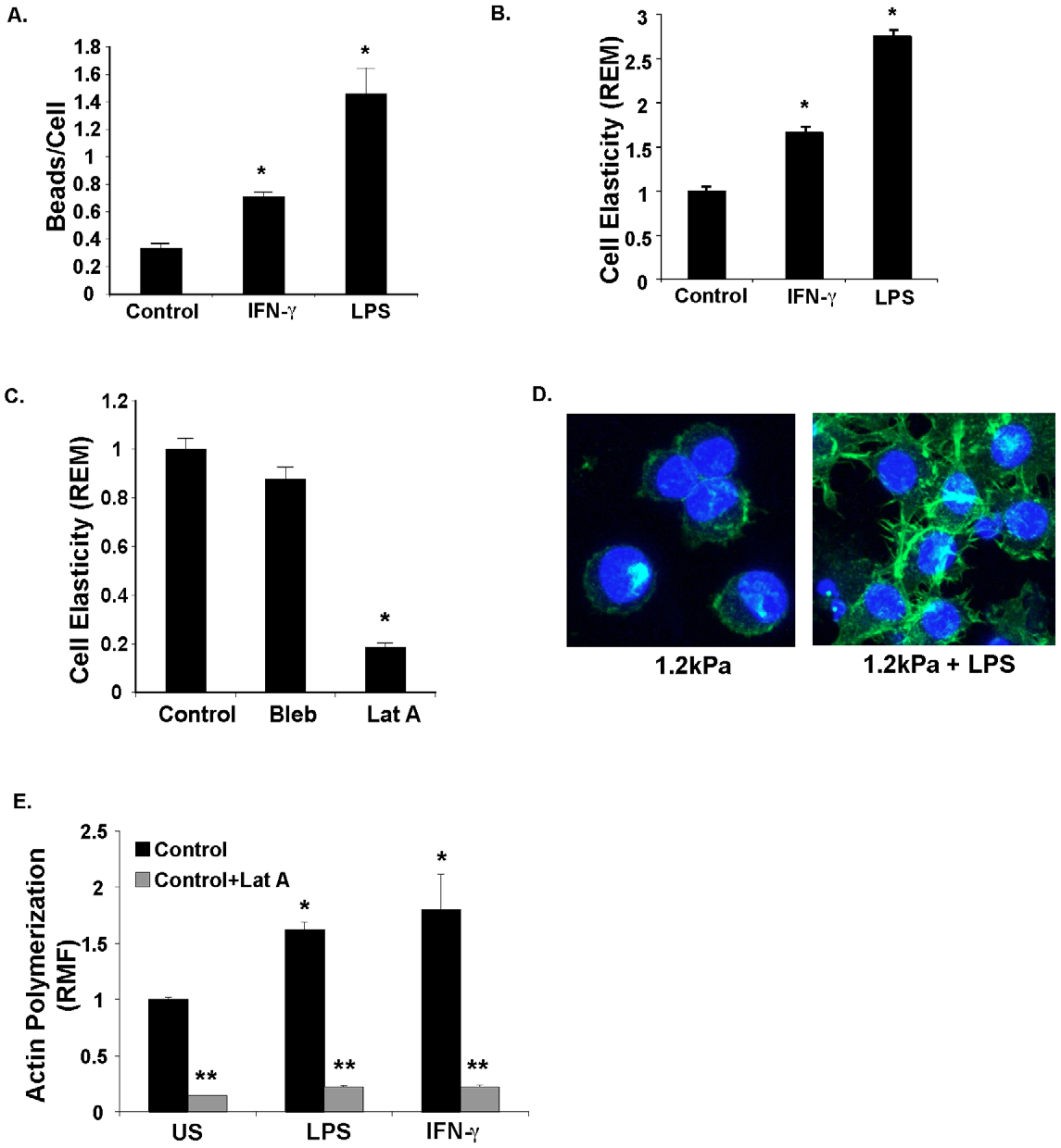
Bacterial PAMPs (LPS) and cytokines (IFNγ) increase macrophage phagocytosis and cell elasticity. **A.** LPS and IFN-γ increase phagocytosis by macrophages on a less rigid (1.2 kPa) substrate in proportion to changes in elasticity. Control, LPS-stimulated (1 µg.ml), or IFN-γ-stimulated (200 U/ml) macrophage were cultured on asubstrate rigidity corresponding to the lungs (1.2 kPa) for 24 hours, and then exposed to IgG-coated latex beads for 2 hours. Beads per cell were manually counted using confocal microscope images. *P = .008 LPS vs. control (n = 5), *P = 0.004, IFN-γ vs control (n = 5) Mann-Whitey U test. **B.** LPS and IFN-γ increase macrophage elasticity on a less rigid (1.2 kPa) substrate. Control unstimulated (US), LPS (1 µg/ml) stimulated, IFN-γ (200 U/ml) stimulated RAW macrophages were cultured on a less rigid substrate for 24 hours, and cell elasticity was measured via Optical Magnetic Twisting cytometry (OMTC). Data is shown as Relative Elastic Modulus (REM). Results depict data from 1 of 3 representative experiments with >100 magnetic beads assayed for each experiment. *P<0.0001 compared to control for both, Mann-Whitney U test. **C.** Macrophage elasticity is decreased by actin polymerization inhibitor. Optical Magnetic Twisting Cytometry (OMTC) measurement of RAW macrophages cultured on more rigid (150 kPa) surface and treated with the myosin inhibitor blebbistatin (Bleb, 50 µM) or the actin polymerization inhibitor latrunculin A (Lat A, 1 µM). Data is shown as Relative Elastic Modulus (REM). Results depict data from 1 of 3 representative experiments with >100 magnetic beads assayed for each experiment. *P<0.001, Mann Whitney U test **D.** LPS stimulation of macrophages on a less rigid (1.2 kPa) substrate increases cell spreading, filapodial projections, actin polymerization. RAW macrophages were cultured on less rigid substrate for 24 hours with or without LPS (1 µg/ml) stimulation for 24 hours. Cells were fixed, stained for polymerized actin (alexa-fluor phalloidin), and DNA (Hoechst nuclear stain), and visualized via confocal microscopy. Images represent collapsed stack of 7 confocal slices. **E.** LPS and IFN-γ increase polymerized actin in macrophages. RAW macrophages in suspension were treated with LPS (1 µg/ml), IFN-γ (200 U/ml), or latrunculin A (1 µM). After 24 hours, cells were fixed, stained for actin with Alexa-fluor phalloidin, and fluorescence was quantified via flow cytometry. Data is show as relative change in mean fluorescence (RMF) from control untreated cells (US). *P = 0.028, n = 4 compared to control, Mann-Whitney U test. **P = 0.028 compared to similar condition without Latrunculin A for each, n = 4, Mann-Whitney U test.

Pathogen clearance and subsequent limitation of inflammation during pneumonia depend upon macrophage phagocytosis [5]. Since phagocytosis requires macrophages to exert physical forces on particles [16], [17], [18], macrophage elasticity may modulate phagocytosis. To examine this possibility, we asked whether biologic agents that stimulate phagocytosis, such as LPS [19] and IFN-γ [20], also increase macrophage cell elasticity. Because substrate elastic modulus (rigidity) has well documented effects on cell elastic modulus (elasticity) [21], [22], [23] (see Supplementary Information S1), we cultured murine RAW 264.7 macrophages on polyacrylamide gels with an elastic modulus, *E*, of 1.2 kPa, which corresponds to rigidity of normal lung tissue [24]. As expected, LPS or IFN-γ increased phagocytosis of IgG-opsonized beads by macrophages compared to untreated macrophages (Figure 1A). Surprisingly, evaluation of cell elasticity by optical magnetic twisting cytometry (OMTC) showed that LPS and IFN-γ treatment each increased macrophage elasticity by 2.7 fold and 1.7 fold respectively, and the magnitude of changes in elasticity correlated with magnitude of change in phagocytosis (Figure 1B).

The observed mechanical changes in macrophages might be attributable to actin polymerization, myosin contraction, or both [25], [26]. To discriminate among these possibilities, murine macrophages were cultured on a more rigid substrate (E = 150 kPa) which corresponds to the rigidity of fibrotic tissue or bone on the high end of the biologic range [27]. Cell elasticity was measured after treatment with a non-muscle myosin inhibitor (blebbistatin) [26] or an actin polymerization inhibitor (latrunculin A) [28] and compared to control. Whereas blebbistatin had minimal effect on cell elasticity compared to untreated cells, latrunculin A significantly reduced elasticity suggesting that in macrophages, the degree of actin polymerization is a major determinant of cell elasticity (Figure 1C). In accordance with elasticity effects, LPS increased spreading and actin polymerization (determined by phalloidin staining) in macrophages cultured on a less rigid substrate compared to untreated macrophages (Figure 1D). Quantification of actin polymerization (determined by phalloidin staining) via flow cytometry confirmed that IFN-γ and LPS each promote actin polymerization (Figure 1D). Thus biologic agents of both bacterial and host origin that promote phagocytosis also increase cell elasticity.

### Substrate rigidity and stretch modulate macrophage phagocytosis through effects on cell elasticity

**Figure 2:**
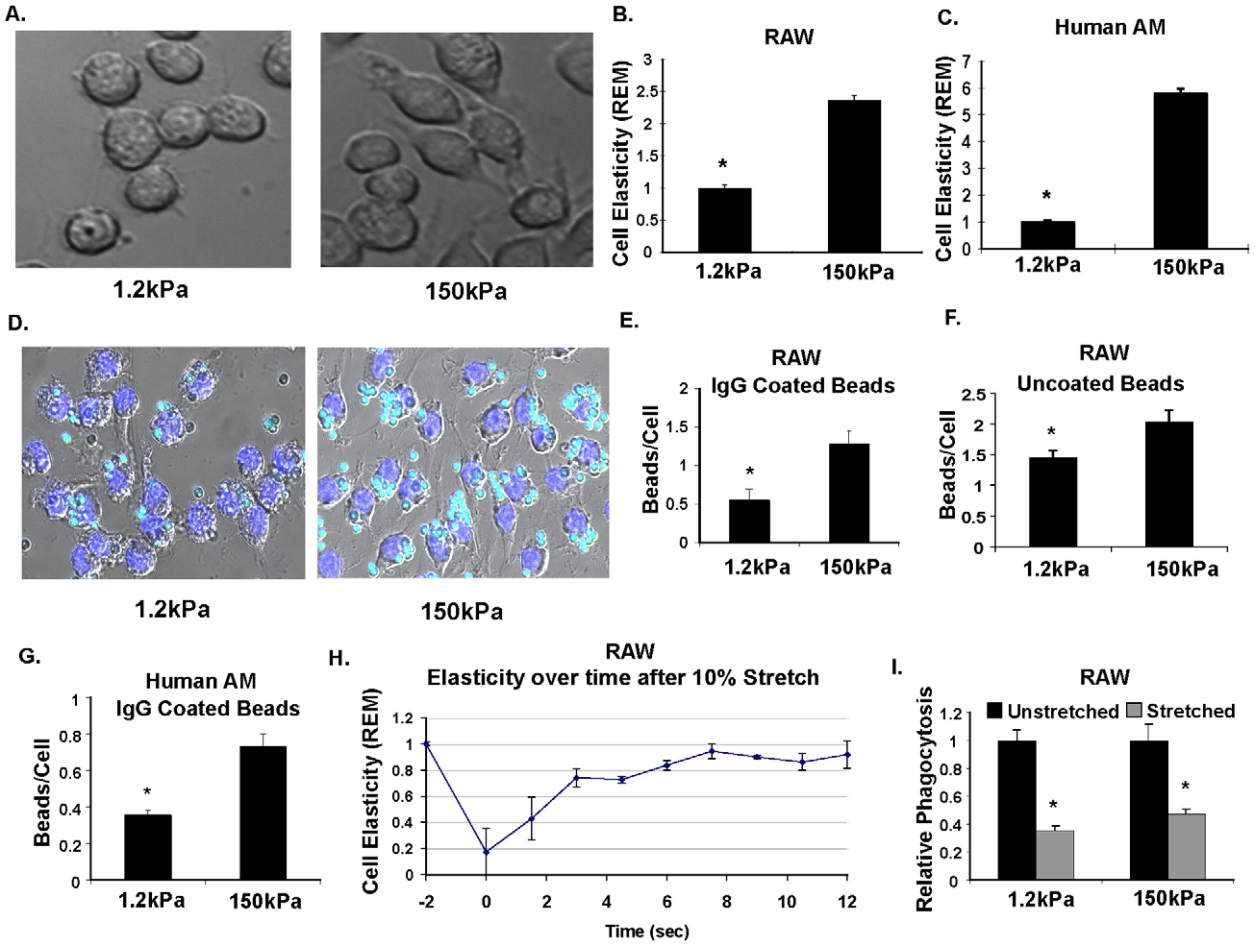
Changes in cell elasticity from physiologic substrate rigidity and stretch modulate macrophage phagocytosis. **A.** Increased cell spreading and filopodial projections of macrophages cultured on a rigid surface. Differential interference contrast (DIC) microscopy images of RAW 264.7 macrophages cultured on a less rigid (*E* = 1.2 kPa) versus rigid (*E* = 150 kPa) polyacrylamide gel coated with poly-L-lysine to facilitate attachment. **B.&C.** Increased elasticity of macrophages cultured on a rigid versus less rigid substrate. Optical Magnetic Twisting Cytometry (OMTC) measurement of mouse RAW macrophages (B.) and human alveolar macrophages (AM) (C.) cultured on less rigid (*E* = 1.2 kPa) versus rigid (*E* = 150 kPa) substrate. Data is displayed as relative elastic modulus (REM) as compared to less rigid condition. *P<0.001 (Mann Whitney U test) Results depict data from 1 of 3 representative experiments with >100 magnetic beads assayed for each experiment. Each experiment had similar results **D.** Increased phagocytosis of beads by macrophages cultured on a rigid versus less rigid surface. Merged DIC and fluorescent images of RAW macrophages cultured on a less rigid versus rigid surface, and exposed to blue-green fluorescent IgG coated latex beads for two hours at standard culture conditions. Unbound beads have been washed away, showing overall fewer beads per cell in soft versus stiff substrate. Nuclei have been stained blue with Hoechst stain. **E&F.** Quantification of phagocytosis in RAW macrophages, counting beads per cell in soft versus stiff macrophages. Results show significantly fewer beads per cell in macrophage cultured on less rigid substrate in both IgG-opsonized (*P = 0.03, n = 5; Mann Whitney U test), and unopsonized (*P = 0.03, n = 5; Mann Whitney U test) beads **G.** Phagocytosis of IgG-coated latex beads is also reduced in human alveolar macrophages (AM) comparing rigid versus less rigid surface. *P = 0.004, n = 5 (Mann Whitney U test) **H.** Substrate stretch decreases macrophage elasticity. Relative elasticity over time after a single 10% stretch of RAW macrophages shows initial 80% reduction in relative elasticity (REM) that returns back to steady state (prestretch) elasticity by about 8 seconds. P<0.0001 for stiffness over time using Spearman Rank Test. Results depict data from 1 of 3 representative experiments with >100 magnetic beads assayed for each time point. **I.** Periodic stretch reduces phagocytosis of uncoated latex beads on either less rigid (1.2 kPa) or more rigid substrate (150 kPa). Results show less phagocytosis (normalized to unstretched cells) in stretched cells on both more rigid and less rigid surface. *P<0.001 (Mann Whitney U test).

We next modulated macrophage elasticity by varying substrate rigidity and stretch and examined the resulting effects on phagocytosis. Either murine (RAW 264.7) or primary human alveolar macrophages (AM) were cultured on a low rigidity substrate (1.2 kPa) which corresponds roughly to rigidity of normal lung tissue [24], or a high rigidity substrate (150 kPa), which corresponds to the rigidity of fibrotic tissue [27]. Appearance of murine macrophages cultured on the less rigid substrate demonstrated a more rounded shape with fewer filopodial projections compared to the more rigid substrate (Figure 2A and Supplementary Information S1). These changes were similar to that observed before and after LPS administration. Murine macrophage elasticity, as measured by optical magnetic twisting cytometry (OMTC), was higher by 2.3 fold for cells on the more rigid substrate compared with the less rigid substrate (Figure 2B), similar to published findings in rat alveolar macrophages [23]. Elasticity of human alveolar macrophages also increased with increased substrate rigidity (Figure 2C). Importantly, the changes in elasticity observed were similar in magnitude to that of the biologic mediators from Figure 1.

Murine macrophages (Figure 2E and 2F) or primary human alveolar macrophages (AM) (Figure 2G and Supplementary Information S1) cultured on the more rigid substrate exhibited increased phagocytosis of both unopsonized and IgG opsonized latex beads compared to cells cultured on less rigid substrate. The magnitude of changes was similar to that observed with LPS or IFN-γ stimulation. Murine macrophages cultured on a more rigid substrate also exhibited increased phagocytosis of bacteria (*M. bovis* BCG) compared to macrophages cultured on a less rigid substrate (Supplementary Information S1). When macrophages on the more rigid substrate were subjected to 10% isotropic biaxial stretch, as would be present in normal lungs, cell elasticity was reduced by 80% (Figure 2H), which subsequently recovered over 5–10 seconds similar to that observed in other cell types [22]. This reduction in cell elasticity through dynamic cyclical stretch also significantly reduced phagocytosis (Figure 2I).

Production of reactive oxygen species (ROS), which is important for antimicrobial activity (Supplementary Information S1), and dynamic membrane protrusions, which are important for the capture of phagocytic targets [16], [17], [18] also increased with increasing substrate rigidity (Video S1, S2, and S3).

Taken together, these data show that macrophage cell elasticity is increased independently and to a similar extent by bacterial PAMPs (LPS), cytokines (IFN-γ), increasing substrate rigidity, or by inhibiting substrate stretch. Moreover, increased cell elasticity, regardless of proximate cause, is associated with increased macrophage phagocytosis.

### Substrate rigidity influences macrophage elasticity through actin polymerization and rhoGTPase activity

**Figure 3:**
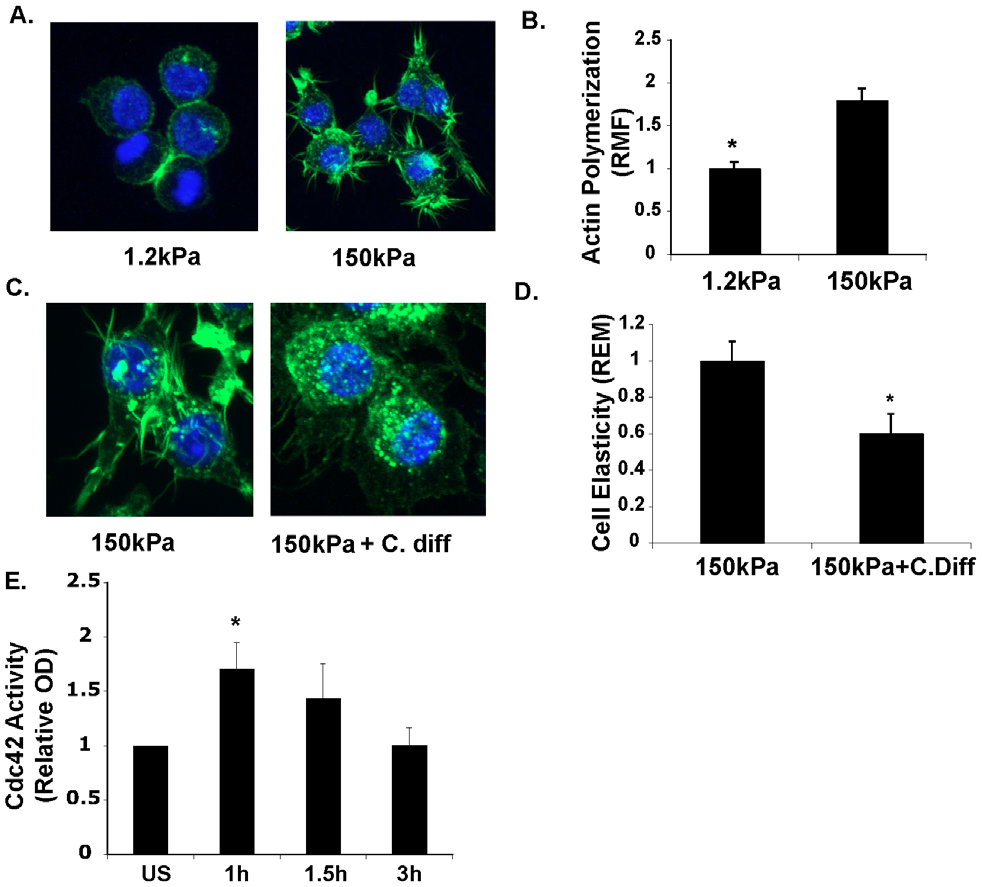
Macrophage elasticity is modulated by actin polymerization and rhoGTPase acitivity. **A.** Decreased polymerized actin staining and filapodia in macrophages cultured on a less rigid surface. Collapsed stack confocal (total of 7 slices) image of RAW macrophages cultured on less rigid (1.2 kPA) or rigid substrate (150 kPa). Green color represents actin staining (alexa-fluor 488 phalloidin) with blue stained nuclei (Hoechst). **B.** Decreased actin polymerization in macrophages cultured on less rigid substrate. RAW macrophages were cultured on less rigid versus more rigid substrate for 24 hours, and then lifted from surface using trypsin/EDTA solution, and immediately fixed, stained for polymerized actin (alexi-fluor-phalloidin), and staining was quantified using flow cytometry. Data displayed as changes in relative mean fluorescence. *P = 0.029, n = 4, Mann-Whitney U test. **C.** Decreased actin-rich filapodial projections and actin fibers in macrophages treated with rhoGTPase inhibitor. RAW macrophages were cultured on more rigid (150 kPa) substrate with or without C. difficile toxin (400 pM) for 24 hours. Cells were then fixed, stained for polymerized actin with alexi-fluor phalloidin and visualized via confocal microscopy. Image represent a collapsed stack of 7 confocal slices. **D.** RhoGTPase inhibitor decreases macrophage elasticity. OMTC measurement of RAW macrophages cultured on more rigid substrate (150 kPa) with or without C. diff toxin (400 pM). *P<0.0001, Mann Whitney U test. Representative experiment for >4 independent observations. **E.** Attachment to substrate leads to activation of cdc42. RAW macrophages in suspension were allowed to adhere to plastic tissue culture dish and protein lysates were assayed for cdc42 activation via ELISA after labeled incubation times. *P = 0.028, n = 4, Mann Whitney U test.

Next, we cultured murine macrophages on soft (1.2 kPa) and stiff (150 kPa) substrates and stained for polymerized actin. Macrophages cultured on the more rigid substrate (150 kPa) demonstrated increased filopodial projections (Figure 3A) compared to macrophages cultured on the less rigid substrate (1.2 kPa). Quantification by flow cytometry confirmed an increase in polymerized actin in cells cultured on a more rigid substrate (Figure 3B). Recognizing that small rhoGTPases (including Cdc42) are important for membrane extension and actin polymerization, we examined the effect of a general rhoGTPase inhibitor, *C. difficile* toxin on cell elasticity and actin architecture. *C. difficile* toxin markedly altered actin architecture (Figure 3C). Specifically, there was an absence of organized actin fibers in filopodial projections. OMTC measurement revealed decreased overall cell elasticity in macrophage treated with *C. difficile* toxin (Figure 3D). Cdc42, which is critical for filopodia formation [29] was specifically activated upon cell adherence to substrate compared to steady state (Figure 3E). Thus increased substrate ridigity, similarly to LPS and IFN-γ, results in increased actin polymerization of macrophages and small rhoGTPases such as Cdc42 promote actin organization and increase cell elasticity.

### Cell elasticity determines macrophage inflammatory response

**Figure 4:**
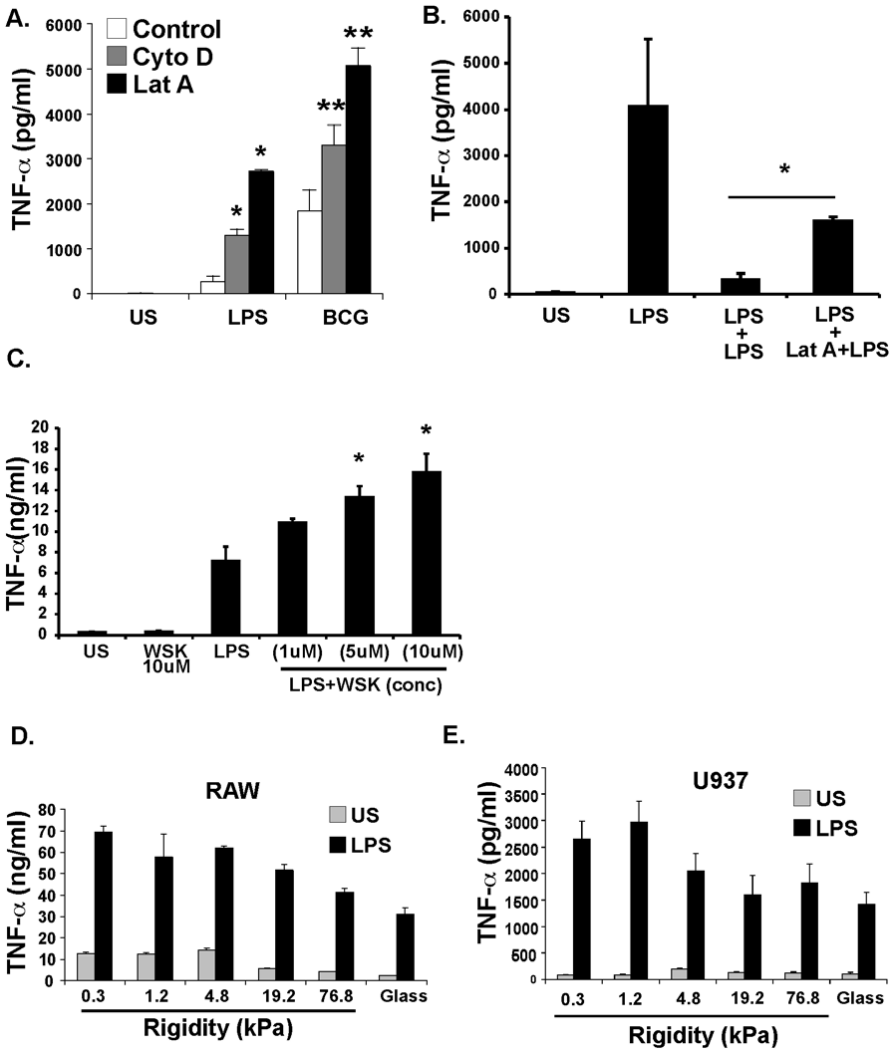
Macrophage elasticity affects response to LPS and LPS tolerance. **A.** Decreasing macrophage elasticity using actin inhibitors increases response to LPS. Human, PMA-differentiated U937 macrophage like cells were cultured on plastic tissue culture plate. Control cells, cells treated with Cytochalasin D (10 µM), or Latrunculin A (1 µM), were cultured for 24 hours with or without LPS (1 µg/ml). Culture supernatants were assayed for TNF-α via ELISA. *P = 0.015 versus control, (n = 6) Mann-Whitney U test. **P = 0.015 versus control, (n = 6), Mann-Whitney U test **B.** LPS tolerance is reduced by actin inhibition on U937 macrophages. Differentiated human U937 macrophages were stimulated with LPS, and had culture supernatant examined for TNFα via ELISA. Levels were compared to cells that were rechallenged with LPS for another 24 hours after 24 hours of initial LPS challenge, or treated Latrunculin A (1 µM) for 30 minutes before rechallenge with LPS. Results show that latrunculin enchanced LPS response with rechallenge. (n = 6) *P = 0.015 **C.** The WASP inhibitor, wiskostatin, increases LPS-mediated TNF-α release. Differentiated human U937 macrophages were stimulated with LPS with or without pretreatment with wiskostatin. Culture supernatants were assessed for TNFα release via ELISA. Results show a dose dependent increase in TNFα release in cells stimulated with LPS with wiskostatin, versus LPS alone. *P<0.01 (n = 6) **D.** Increasing substrate rigidity decreases TNF-α release in response to LPS in RAW macrophages. RAW macrophages were cultured in a 96 well plate in wells containing poly-L-lysine coated gels of increasing elastic modulus from 0.3 kPa-76.8 kPa, or on poly-L-lysine coated glass for 24 hours. Cells were then stimulated with LPS (1 µg/ml) for 24 hours and culture supernatants were assayed for TNF-α via ELISA. P<0.001 for correlation of TNF level with substrate rigidity. Spearman Rank Test. **E.** Increasing substrate rigidity decreases TNF-α release in response to LPS in human U937 macrophage-like cells. Protocol similar to D, except PMA-differentiated U937 cells are used. P<0.01, Spearman Rank Test.

Having observed that LPS increases macrophage cell elasticity, and considering that LPS stimulation causes hyporesponsiveness to subsequent LPS exposure or tolerance [30], we hypothesized that increased cell elasticity would decrease LPS responsiveness. Because murine macrophages showed high levels of toxicity after prolonged exposure to actin inhibitors, we examined the effect of actin inhibitors in human U937 macrophages. Both cytochalasin D, and latrunculin A, which have distinct mechanisms of actin polymerization inhibition [28], significantly increased LPS-mediated (and BCG-mediated) TNFα release compared to control macrophages cultured on a rigid substrate (Figure 4A). Moreover, inhibiting actin polymerization also reduced macrophage tolerance to LPS re-stimulation (Figure 4B). In order to determine if targeting actin polymerization by another mechanism could also affect response to LPS, we examined the role of the Wiskott-Aldrich Syndrome protein (WASP). WASP is a critical mediator of actin assembly, is activated by Cdc42, and WASP mutations lead to impaired macrophage function in Wiskott-Aldrich Syndrome [31]. Incubation of human macrophages with wiskostatin, a WASP inhibitor [32], also increased macrophage TNFα release in response to LPS (Figure 4C).

To determine if modulating cell elasticity by changing the substrate rigidity produced similar effects as actin inhibitors on LPS response, murine macrophages or differentiated human U937 macrophages were cultured on a 96 well plates lined with polyacrylamide gels over a physiological range of elastic moduli (0.3 kPa to 76.8 kPa), or extremely high elastic modulus (glass with no gel). With LPS stimulation, TNFα release decreased with increasing substrate rigidity (Figure 4D, 4E) in both macrophage cell types. Taken together, these data demonstrate unanticipated roles for macrophage elasticity and actin polymerization in macrophage response to LPS and LPS tolerance. Moreover using actin polymerization as a target, we were also able to identify the novel role of WASP in determining macrophage response to LPS.

### Cell elasticity modulates macrophage transcriptomic profiles

**Figure 5:**
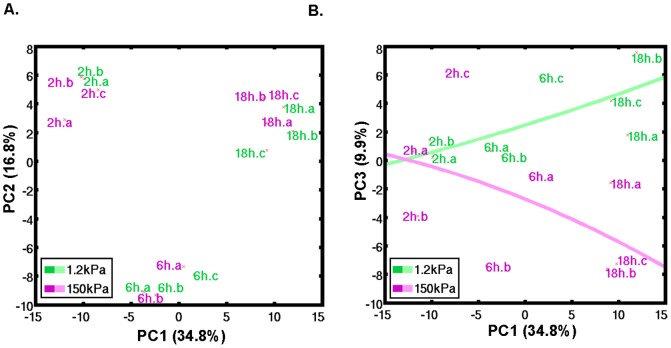
Macrophage gene expression is determined by duration of culture on substrate and substrate rigidity. Gene expression profile over time in RAW macrophages increasingly correlates with substrate rigidity. RAW macrophages, after removing from a plastic tissue culture dish, were plated on less rigid (E = 1.2 kPa) or more rigid (E = 150 kPa) substrate for 2 hours, 6 hours, and 18 hours, and RNA was extracted for microarray analysis at each time point. **A.** Principle component analysis (PCA) reveals a PC1 that well correlates with duration of culture on substrate and describes the largest variance (34.8%), while a PC2 distinguished 6 hours culture time from 2 hours and 18 hours culture times. **B**. The third larges source of variance was PC3 (9.9% of variance) that distinguishes by substrate rigidity increasingly over time.

To determine whether changes in elasticity modulate transcriptomic profiles of macrophages, we compared murine macrophages cultured on a less rigid (1.2 kPa) versus more rigid (150 kPa) substrate over 2–18 hours using microarray analysis. Principal component (PC) analysis of the transcriptomic profiles revealed that PC1 accounted for 34.8% of the variance, PC2 for 16.8% of the variance, and PC3 for 9.9% of the variance. The specific time points of culture were well distinguished along PC1 (see Figure 5A). Along PC2 the 2 hour and 18 hour time points showed similar directions of variance, while the 6 hour time point was distinguished between these two (Figure 5A), suggesting there were specific changes in gene activation at 6 hours culture time. Significantly, PC3 distinguished macrophage groups by substrate rigidity, and this separation although limited at 2 hours, increased at 6 hours, and further increased at 18 hours (Figure 5B). At 18 hours, there were 24 genes significantly up regulated and 34 genes significantly down regulated in macrophages on a more rigid as compared to less rigid substrate (Supplementary Information S1). Surprisingly, analysis revealed no significant enrichment in phagocytosis or inflammation related gene ontologies. These data suggest that although macrophage gene expression may change over time according to substrate rigidity, gene ontology analysis does not readily predict observed effects of rigidity on phagocytosis or LPS response.

## Discussion

These data show that cell elasticity is a critical determinant of macrophage innate function. Cell elasticity is modulated not only by classical biologic activators such as LPS and IFN-γ, but to an equal extent by substrate rigidity and substrate stretch. Consequently, macrophage elasticity may be a common pathway by which mechanical and biologic stimuli regulate specific macrophage functions such as phagocytosis or LPS responsiveness. Moreover, specifically targeting macrophage elasticity to modulate macrophage function may provide novel therapeutic targets, such as using WASP function to modulate macrophage inflammatory response to LPS.

In lungs and other vital organs, alteration of tissue rigidity has been regarded as a mere by-product of injury and inflammation [11], [33], [34], [35]. Moreover, bacterial PAMPs or cytokines increase lung elastic modulus both at the organ level [12], [13] and at the cellular level [14], [15] although the influence of these changes on cellular function is not well understood. Where regulation of macrophage function can determine the outcome of injury and inflammation [1], [2], [3], prevailing theories have focused on receptor-ligand dependent modulation of macrophage activation and function [1], [2], [3] and its downstream consequences in terms of gene expression [1], [8], [36]. Specifically, prior exposure to cytokines, PAMPs, or both determine subsequent macrophage response to stimuli characterized by “classical (M1)” or “alternative (M2)” macrophage activation [2] although there may be many other macrophage activation phenotypes [1]. Our findings show that in addition to changes in gene expression, PAMPs and cytokines promote changes in macrophage cell elasticity, which have specific effects on macrophage function. These findings suggest that the increased tissue rigidity that often accompanies pneumonia or other types of inflammation in combination with increased cell elasticity promoted by PAMPs or inflammatory cytokines increase macrophage phagocytosis while decreasing the inflammatory response, which may serve to limit organ damage and promote resolution of inflammation.

Substrate rigidity influenced macrophage transcriptomic profiles and changes in the transcriptome were related to both duration of culture on specific substrate and substrate rigidity. In contrast to prior attempts to characterize macrophage phenotype by gene expression [1], [8], [36], observed changes in macrophage gene expression did not readily predict our observed changes in phagocytosis and inflammatory cytokine release associated with differing substrate rigidity. This would suggest that cell elasticity-induced changes in macrophage function may occur by alternate mechanisms such as post-translational protein modifications, although novel gene pathways or genes not identified on microarray analysis may also be responsible for the changes observed. Post-translational modifications affecting LPS responsiveness may include, for example, cell elasticity-associated effects on TLR-ligand complex uptake, which can control LPS responsiveness [37]. Changes in phagocytosis may be related to degree of cell spreading and membrane extension that were observed with changing cell elasticity (by either substrate rigidity or LPS/IFN-γ stimulation) resulting in increased cell surface area for particle binding and phagocytosis.

In summary, our findings suggest that macrophage elasticity determines macrophage innate function. Furthermore, macrophage elasticity is modulated independently by bacterial PAMPs (LPS), cytokines (IFNγ), physiologic substrate rigidity and physiologic substrate stretch, and is dependent on actin polymerization and small RhoGTPase activity. The unexpected finding that cell elasticity exerts a dominant influence on macrophage function represents an underappreciated factor that defines macrophage function. Traditional in vitro systems making using of static plastic or glass surfaces for cell culture would overlook these factors. Furthermore, the observation that cell elasticity determines macrophage function in cells of different sources and species suggests these findings may have broad application to macrophage molecular cell biology in a variety of disease states characterized by increased tissue rigidity and altered macrophage function such as, atherosclerosis [38], [39], invasive tumors [3], [40] or fibrotic tuberculosis [41]. These findings advance our current understanding of factors that influence fundamental macrophage function. Moreover, strategies targeting macrophage elasticity may lead to novel treatments for human diseases.

## Methods

### Macrophage cell lines

Experiments used RAW 264.7 mouse macrophages or human promonocytic U937 (American Tissue Cell Company, ATCC). RAW cells were cultured in complete DMEM medium (10% heat-inactivated FCS), lifted off with trypsin-EDTA (GIBCO) and transferred to polyacrylamide gels for 24 hours before use. U937 cells were cultured in complete RPMI 1640 medium (10% heat-inactivated FCS). U937 cells were PMA differentiated as published [42].

### Human Alveolar Macrophages

Healthy individuals without evidence for active pulmonary disease and with normal spirometry were prospectively recruited. Lung immune cells were obtained by BAL using standard technique [42]. All procedures were performed on adults after informed consent following protocols approved by the Beth Israel Deaconess Medical Center Institutional Review Board. The cells were separated from pooled BAL fluid and AM were isolated by adherence to plastic-bottom tissue culture plates as previously described [42].

### Reagents

Latrunculin A and Cytochalasin D were obtained from EMD Biosciences (San Diego, CA). Cells were preteated with Latrunculin A (0.1–10 µM) or Cytochalasin (5 µM) at least 30 minutes before downstream assay. LPS (1–10 µg/ml) was obtained from Sigma-Aldrich St. Louis, MO). Mouse recombinant IFN-γ (200 U/ml) was obtained from Antigenix America Inc (Huntington Station, NY). Alexafluor-488 phalloidin from Invitrogen (Carlsbad, CA). Human and mouse IgG was obtained from Jackson Immunoresearch (West Grove, PA).

### Polyacrylamide Gel Preparation

Gel substrates were prepared according to a previously described protocol [25]. Concentrations for 1.2 kPa:5% Acrylamide and 0.03% Bis-acrylamide and for 150 kPa:20% Acrylamide and 0.2% Bis-acrylamide. Following gelation, the surface was activated with 200 µL solution of 1 mM Sulfosuccinimidyl-6-[4-azido-2-nitrophenylamino] hexanoate (Sulfo-SANPAH; Pierce, Rockford, IL), coated with poly-L-lysine. Cells were allowed to adhere overnight on gels before performance of downstream experiments.

### Cell Mapping Rheometry (CMR)

Biaxial deformation was imposed on an elastic polyacrylamide substrate using a novel punch indentation system as previously described [25] which provides a uniform biaxial strain that can be applied repeatedly to produce physiologic cell strains.

We measured cell rheology using OMTC as previously described [22]. Briefly, poly-L-lysine-coated ferrimagnetic beads are allowed to bind to the cell surface receptors [22] and become tightly anchored to the cytoskeleton through focal contacts. The beads are permanently magnetized in the horizontal plane of the cell culture and subsequently twisted in an oscillatory magnetic field with frequency 0.75 Hz. The twisting field causes each bead to rotate towards alignment with the oscillatory field and, as a result, a weak mechanical torque is applied to the cell. The complex modulus of the cells (G*) is computed from the Fourier transform of the applied mechanical torque (*T*) and of the resulting lateral bead displacement (*D*):
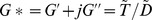
where * denotes a complex number, the tilde overbar denotes the Fourier domain, and *j* ^2^ = −1. For each experimental condition, data are reported as medians of the bead populations (215–719 beads on a similar number of cells per experimental condition). Because macrophages are phagocytic cells and internalization of beads would result in increased stiffness measurements due to artifact, we first determined that the great majority of beads (>95%) could still be washed off of macrophages at less than 20 minutes incubation implying that beads were not yet internalized. Using this metric, all OMTC measurements were conducted with <20 minutes incubation time with the beads to prevent experimental error associated with bead internalization.

### Phagocytosis assay

Macrophages were incubated with fluorescent latex beads (2 µM diameter; Polysciences, Inc., Warington, PA) at a multiplicity of 5∶1(beads/macrophages) for 2 hours at 37°C in 5% CO2. Noningested beads were removed by washing, and the adherent cells were fixed in 4% paraformaldehyde for 15 minutes. For each determination performed in duplicate (and total of three independent experiments), the number of beads associated per 200 macrophages was counted on at least two separate gels. Phagocytosis was measured by Leica confocal microscope via water emersion lens and reported as phagocytosed beads per macrophage or percentage of cells uptaking particles. Beads were coated with mouse or human IgG using manufacturer's protocol (Polysciences). Only those beads that were clearly within the cytoplasm at the nuclear slice were considered internalized. To visualized beads we used a GFP-BCG kindly provided by Dr. Deborah Hung at the Broad Instititute [42].

### Actin polymerization assay

Cells in suspension were treated with LPS (10 µg/ml), IFN-γ (200 U/ml), with or without latrunculin A (1 µM), for 24 hours. Cells were then fixed with 4% formaldehyde, permeabilized with 0.1% Triton-X in PBS, and stained with either rhodamine-phalloidin (Sigma-Aldrich, St. Louis, MO) or Alexa-fluor 488 phalloidin. (Invitrogen). Results were then analyzed via Beckman Coulter FC500 flow cytometery. For studies involving adherent cells, cells were lifted from gels with Trypsin-EDTA and immediately put in 4°C fixative with 4% formaldehyde to prevent any actin rearrangement. Cells were then stained and analyzed. For confocal microscopy images, cells cultured on gels were fixed and stained with Alexa-fluor 488-phalloidin. Cells were imaged with a 40× water immersion lens on a Leica confocal microscope. 7–10 slices were obtained and images depicted represent a collapsed stack image.

### RhoGTPase assay

Cdc42 activation was measured using the G-LISA Small G-protein activation assay according to manufacturer's protocol (Cytoskeleton, Inc., Denver, CO). Cells were allowed to adhere to rigid substrate (150 kPa) for 0–3 hours at standard culture conditions. Culture media was aspirated, and protein was extracted using lysis buffer. Non-adherent cells were obtained from aspirated media via centrifugation, and protein lysate from these cells was combined with that of adherent cells to assay Cdc42 activation of total cell population (adherent and loosely adherent cells).

### Cytokine detection

Cells were cultured overnight on a 96 well system with plate bottoms having variable stiffness polyacrylamide gels coated with poly-L-lysine. Cells were then stimulated with LPS (1 µg/ml) for 24 hours, and culture supernatant was assayed for TNF-α via ELISA (R&D Systems, Minneapolis, MN). Cells were not washed prior to assay to assure uniform number of cells per well. Cell number was not significantly different in low versus high rigidity substrate wells when assayed via Hoechst staining.

### Reactive oxygen species assay

RAW264.7 cells were plated 100,000 per well in a 96 well plate. After overnight culture, 100 µL of 10 uM 2′,7′-dichlorodihydrofluorescein diacetate (Molecular Probes (now Invitrogen, Carlsbad, CA)) working solution diluted in HBSS was placed in each well. Each well was then loaded with IgG coated latex beads at an MOI of (20∶1). The control well was loaded with HBSS alone without beads. Oxidative burst was measured using excitation wavelength of 490 nm and emission at 525 nm for 0, 20, 40, 80, and 120 minutes. Confirmation of cell number per well was assayed using Hoechst nuclear staining. It showed no differences in results when corrected for cell number.

### Microarray Analysis

For transcriptional profiling, the mouse genome 430 2.0 Affymetrix GeneChip, containing more than 45,000 transcripts was used. RNA for the microarray experiments was obtained from each of three independent experiments using RAW macrophages cultured upon a soft (1.2 kPa) versus a stiff (150 kPa) surface for 0–24 hours. RNA was extracted according to manufacturer's protocol (RNeasy, Qiagen, Germantown, MD). Microarray analysis was conducted via the Genomics Center at the Beth Israel Deaconess Medical Center. cDNA synthesis, *in vitro* transcription reaction for production of biotin-lableled RNA, hybridization of cRNA, and scanning of output image file was performed as previously described [43].

Expression values were extracted from.CEL files using Robust Multi-array Average (RMA, for specific sample subsets) and Microarray Suite 5.0 (MAS 5.0) separately using BioConductor (http://www.bioconductor.org). Geometric fold method was used to assess the genes that change significantly in their expression between soft and stiff substrates [44]. The data were analyzed further with principle component analysis [44]. Microarray data has been uploaded and is available at http://www.ncbi.nlm.nih.gov/geo under data set GSE36878.

### Statistical Analysis

The non-parametric Mann-Whitney U test was used to compare groups with continuous variables such as beads/cell. A non-parametric Spearman's Rank Correlation was used to compare two non-dichotomous variables such as multiple substrate stiffnesses and TNF-α release. A value of p<0.05 was considered significant. Fisher's exact test was used for 2×2 tables for the microarray analysis.

## Supporting Information

Video S1 **Murine macrophages cultured on a less rigid (1.2 kPa) substrate exhibit spontaneous dynamic extensions.** (See arrows).
(MOV)

Video S2 **Murine macrophages cultured on a more rigid (76.8 kPa) substrate exhibit relatively longer spontaneous dynamic extensions (see arrows) compared to less rigid substrate suggesting larger sampling area for phagocytosis.**
(MOV)

Video S3 **Murine macrophages cultured on a more rigid (76.8 kPa) substrate.** Movie demonstrates capture of a two beads via membrane projection and subsequent phagocytosis.
(MOV)

Supplementary Information S1 (DOC)
