# Supplementary material for: Cell Elasticity Determines Macrophage Function
Source: PLoS One. 2012 Sep 18;7(9):e41024. doi: 10.1371/journal.pone.0041024 (PMC3445606; doi:10.1371/journal.pone.0041024)
Supplement: Supplementary Information S1 — (DOC) [file pone.0041024.s004.doc]

**Supplemental Data and Information:**

**I. Culture of macrophages on polyacrylamide gels.** Macrophages were cultured on polyacrylamide gels of varying elastic modulus (rigidity) and then coated with poly-L-lysine to facilitate attachment as detailed in the methods section. Macrophages in general appeared more rounded with less spreading on less rigid substrate (1.2kPa) versus more rigid substrate (150kPa) (Figure 1A). In addition to cell morphology, degree of attachment of the macrophages on the less rigid substrate was decreased (Figure S2) and cells were more easily detached from substrate. Furthermore the addition of periodic substrate stretch decreased attachment further. Alveolar macrophages (AM) can be loosely adherent and easily recovered via bronchoalveolar lavage[1] whereas subpopulations may remain even after lavage[1]. Adherence for >90% of these loosely adherent AM recovered via lavage in vitro to plastic tends to be firm[2]. Our observations in vitro that macrophages adhere less firmly to less rigid substrates and with periodic stretch may therefore model degree of attachment observed in vivo. Furthermore, AM in vivo are phagocytic[3], although lung in vivo phagocytic capacity may be limited in resting AM compared to activated AM[4]. Our findings demonstrate that modulation of macrophage elasticity by either substrate rigidity or biologic mediators (LPS or INF-γ), influence macrophage phagocytic capacity.


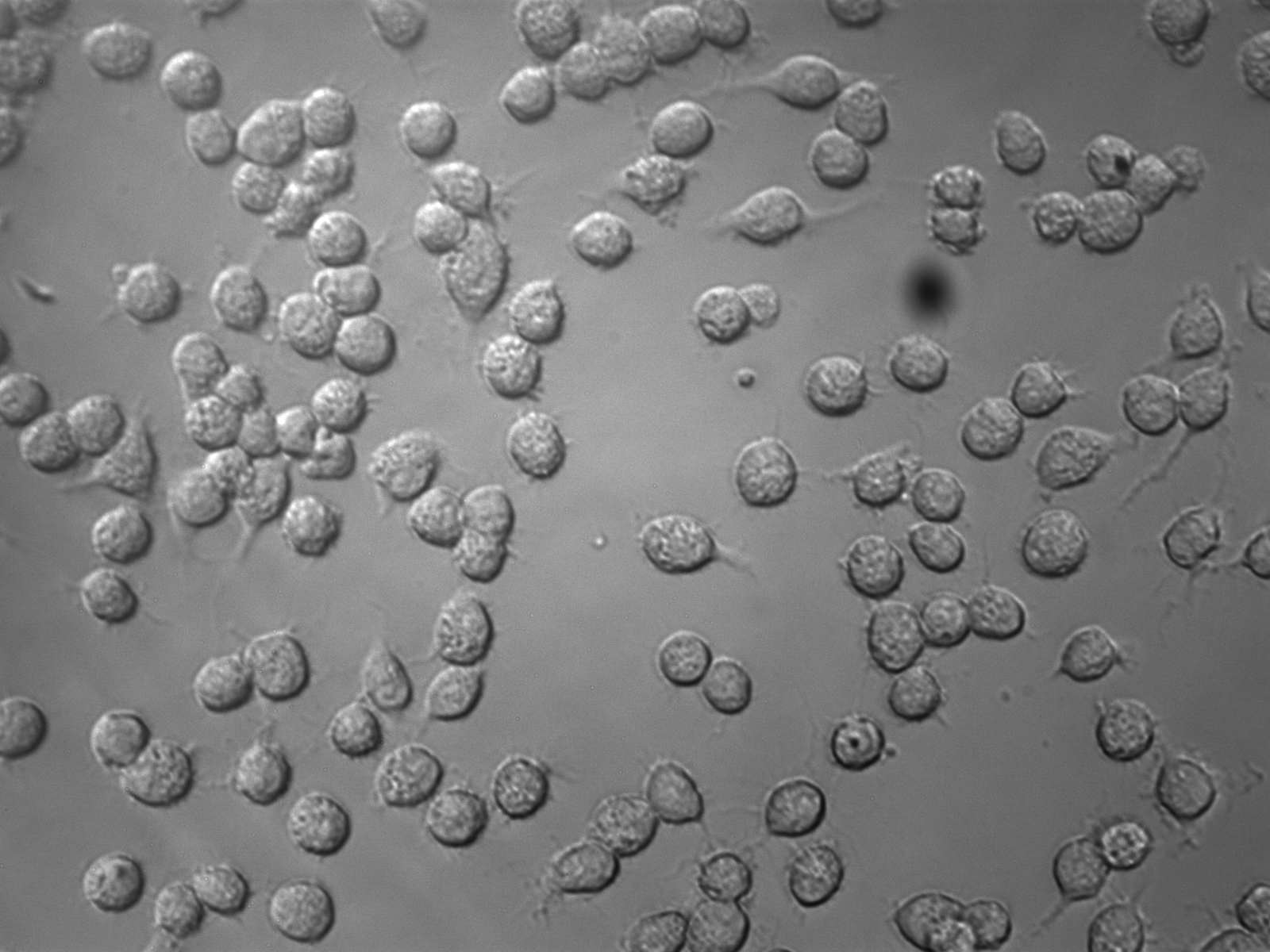

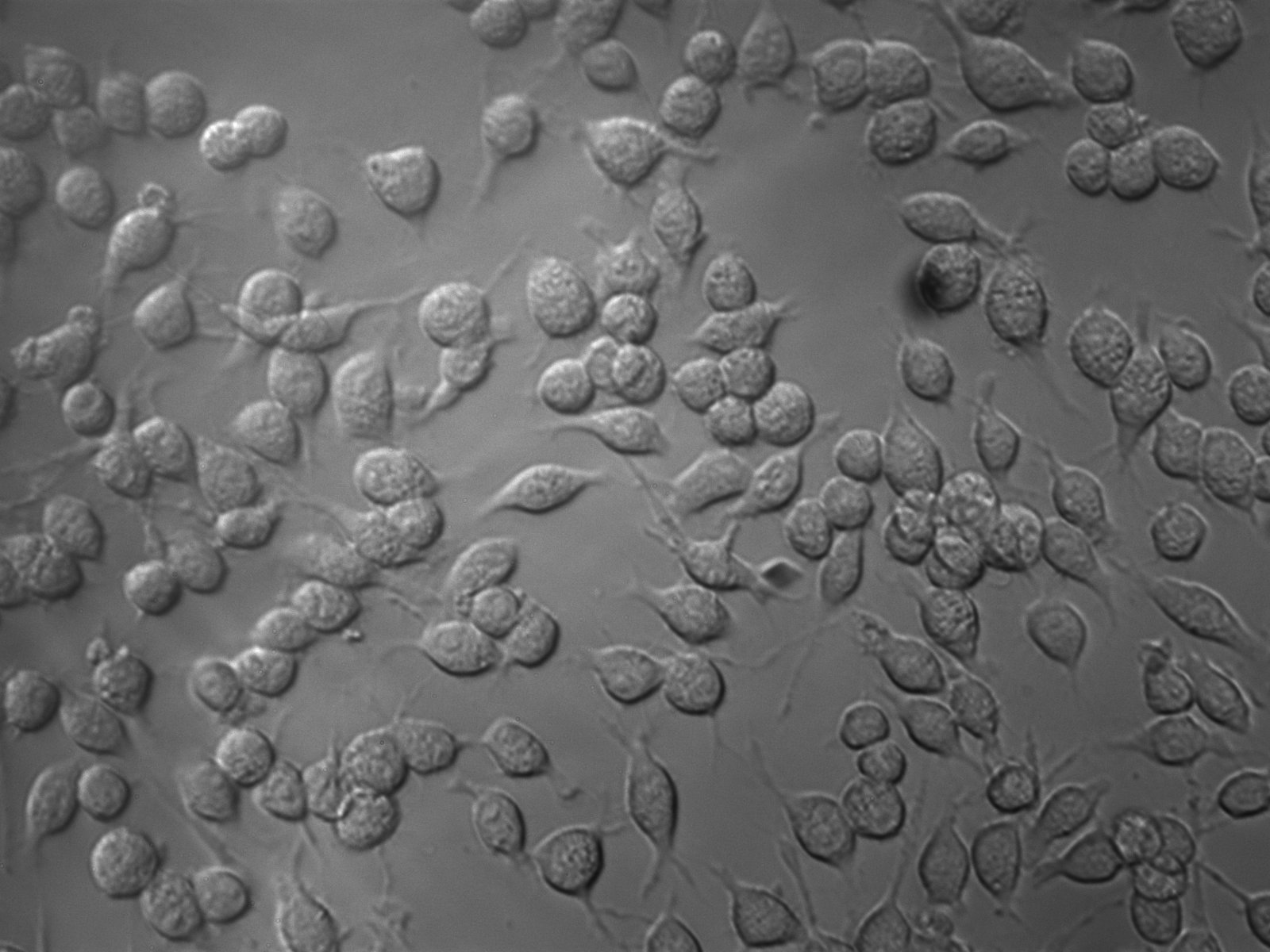


**1.2 kPa**

**150 kPa**

**Figure S1: Decreased macrophage density and adherence on less rigid substrate.** Murine RAW cells cultured on less rigid substrate demonstrate decreased numbers of attached cells and light washing would further reduce numbers of attached cells(not shown).

**II. Elastic Modulus.** Elastic modulus is defined as the resistance of the constituent material of an object to deformation by an applied force independent of object shape or dimensions. For example if material had a given force *F* applied to it, the material would deform a certain length *l*. The applied force F can be normalized to the cross-sectional area of the material (by dividing F by the area) to obtain the applied stress σ. The deformation Δ*L* can also be normalized to the reference length of the object to obtain strain (Δ*L*/*L*). The elastic modulus *E* therefore is calculated as the ratio of stress to strain and since strain is dimensionless, *E* is expressed as a unit of force (pascals or Pa). For clarity, cell elastic modulus has been referred to as “elasticity” while substrate elastic modulus is referred to as “rigidity”, although they represent identical measures for different materials (cell vs. substrate). Elasticity values for cells in this study are displayed as relative values to control in order to combine results from independent experiments.

**III. Effect of substrate rigidity on phagocytosis of *M. bovis* BCG.** RAW 264.7 macrophages were cultured overnight on a less rigid (*E*=1.2kPa) or more rigid (*E*=150kPa) substrate, and then exposed to GFP-expressing *M. bovis* BCG for 2 hours (5:1 MOI). Because of difficulty counting individual particles, data was collected as percent of total cells that had internalized GFP-expressing BCG anywhere within the confocal slices of the cell. (GFP-BCG was a gift from Deborah Hung, MD, PhD, Broad Institute). Results show increased percentage of AM with uptake of BCG when cultured on more rigid substrate versus less rigid substrate (65% vs 50%, P=0.008, n=5, Mann-Whitney U test).

*

**(% positive) cells)**

**Figure S2. Phagocytosis of *M. bovis* BCG is decreased with decreased matrix stiffness**

**
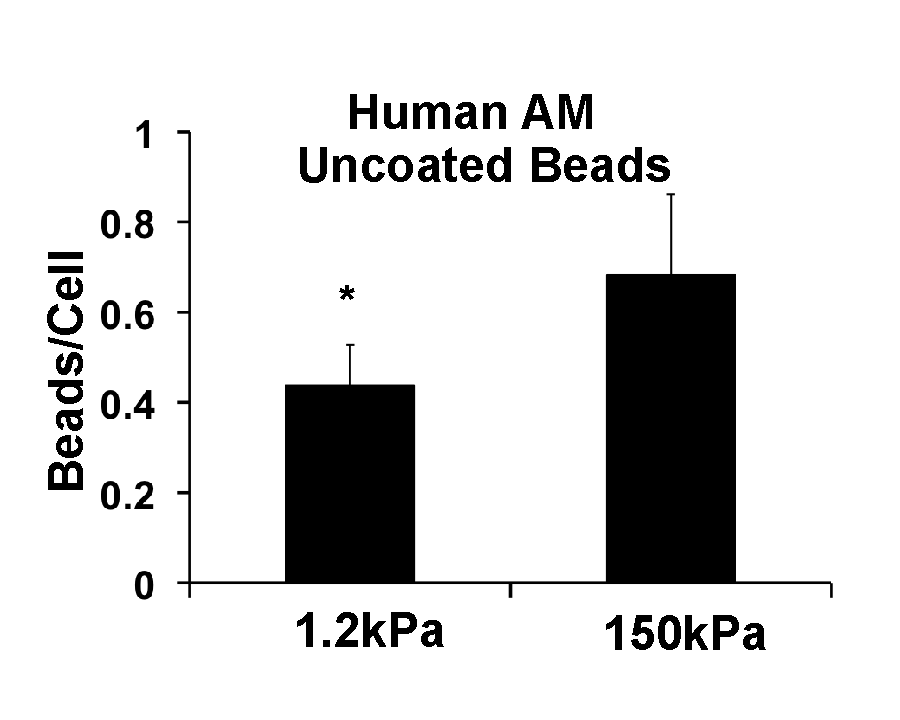
**

**Figure S3. Phagocytosis of uncoated latex beads in human AM is decreased with decreased matrix stiffness**

**IV. Phagocytosis of uncoated latex beads by human AM.** Human alveolar macrophages (AM) were cultured overnight on a less rigid (*E*=1.2kPa) or more rigid (*E*=150kPa) substrate, and then exposed to uncoated 2μm latex beads for 2 hours (5:1 MOI). Total number of beads per cell were calculated. Results in Figure S3 show increased uptake of beads in AM cultured on a more rigid substrate versus less rigid substrate (0.68 beads/cell vs 0.43 beads/cell, P=0.04, n=6, Mann-Whitney U test).


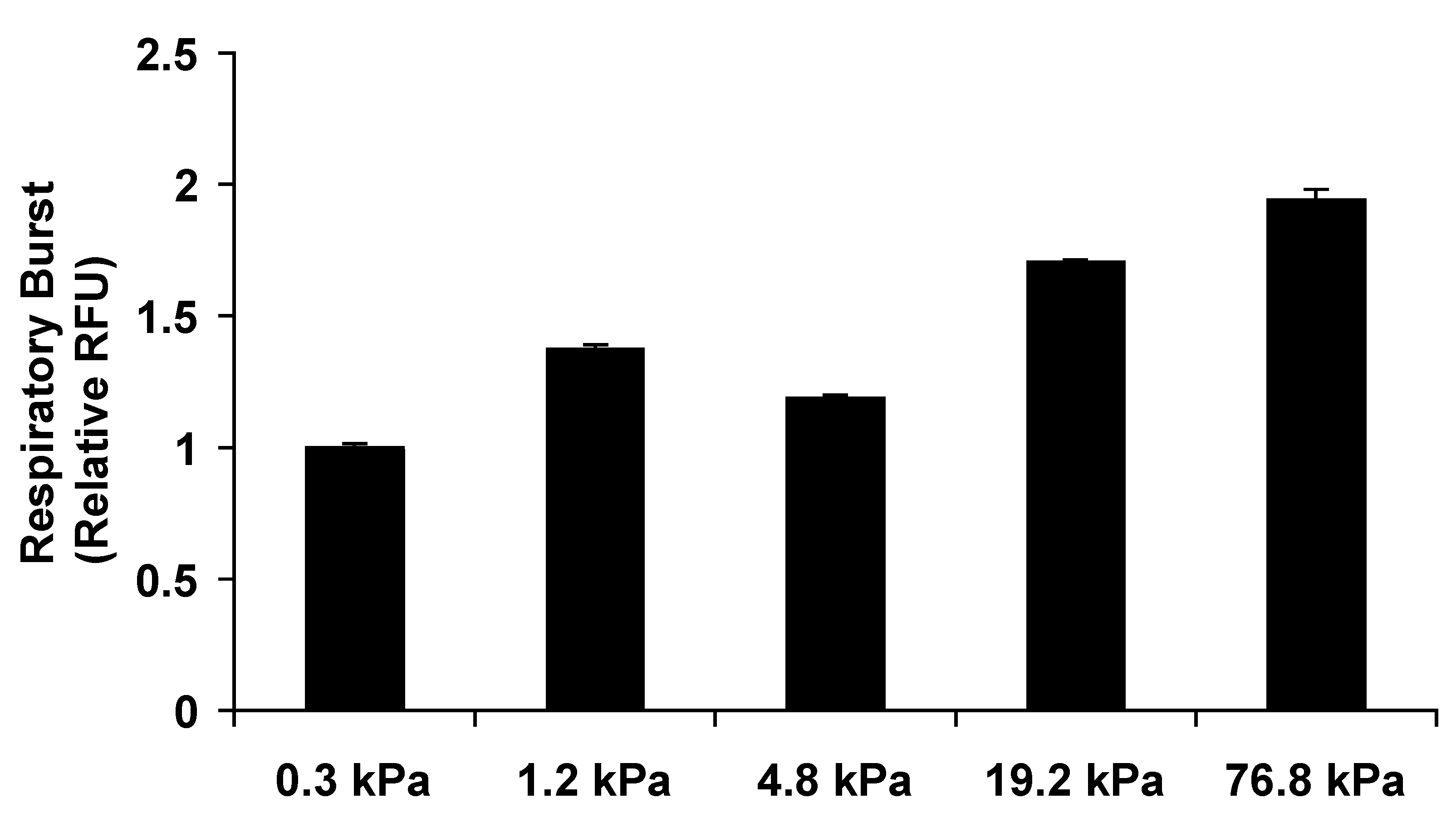


**Figure S4.** Reactive oxygen species production increases with increasing stiffness.

**V. Reactive Oxygen Species Assay.** In order to determine if substrate rigidity modulates respiratory burst, we assayed respiratory burst using dichlorodihydrofluorescein (DCFH, (Molecular Probes (now Invitrogen, Carlsbad, CA))). RAW macrophages (1x10^5^/well) were cultured on a range of substrate stiffnesses using a 96 well plate with wells covered poly-L-lysine coated gels of increasing elastic modulus from 0.3kPa-76.8kPa. Cells were exposed to IgG-coated latex beads (MOI 20:1) for 0-120 minutes and fluorescence was assayed using a fluorescent microplate reader (excitation wavelength of 490nm and emission at 525nm) at a number of time points from 0-120minutes. Figure S4 shows results at 20 minutes showing significant correlation (P<0.001) (n=4 for each rigidity) between amount of reactive oxygen species produced and substrate stiffness. Spearman Rank Correlation Test. Data represents mean of 4 independent observations. Similar differences were observed throughout the course of the experiment.

**Vl. Dynamic membrane extension of macrophages.** Recent studies suggest that during phagocytosis, the act of particle binding is not a passive event, but requires membrane extension and capture of particles by macrophages[5]. This membrane extension occurs constitutively and randomly, suggesting that the macrophage is constantly “probing” its environment for phagocytic targets[5]. Other studies have demonstrated that phagocytosis seems to occur after this type of binding by retraction of phagocytic tentacles that are attached to particles[6,7]. Consequently, this would suggest that increased area of sampling by macrophages would increase phagocytosis. We observed live resting macrophages (RAW 264.7) cultured on either a less rigid (1.2kPa) or more rigid substrate (76.8 kPa), and obtained multiple images over time (at a rate of 1 image ever 6 seconds for 10 minutes). The video (Movie 1) shows that cells on the less rigid surface tended to be more rounded and extended short projections (see arrows). The cells on the rigid surface were more spread out with more dynamic membrane extensions suggesting a larger sampling area (Movie 2). Dynamically imaging macrophages cultured on a more rigid surface (76.8kPa) during phagocytosis demonstrate how dynamic membrane extensions capture phagocytic targets (Movie 3). Thus macrophages cultured on a more rigid substrate have a larger area of probing or sampling, and this may, in part, explain the mechanism by which phagocytosis is increased in macrophages cultured on a more rigid versus less rigid substrate.

**Movie 1:** Murine macrophages cultured on a less rigid (1.2kPa) substrate exhibit spontaneous dynamic extensions. (See arrows)


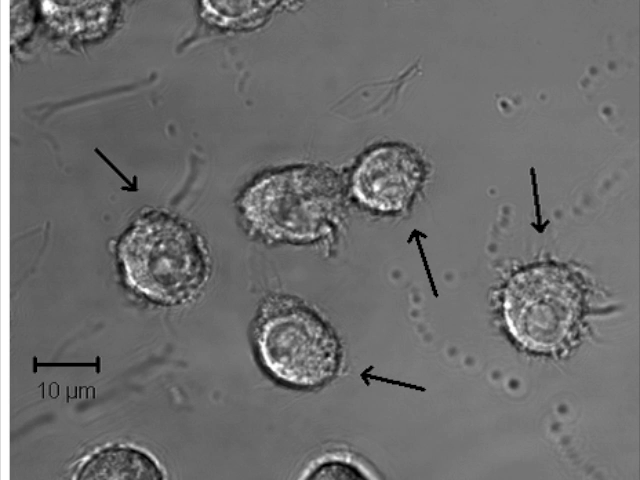


**Movie 2:** Murine macrophages cultured on a more rigid (76.8kPa) substrate exhibit relatively longer spontaneous dynamic extensions (see arrows) compared to less rigid substrate suggesting larger sampling area for phagocytosis

**
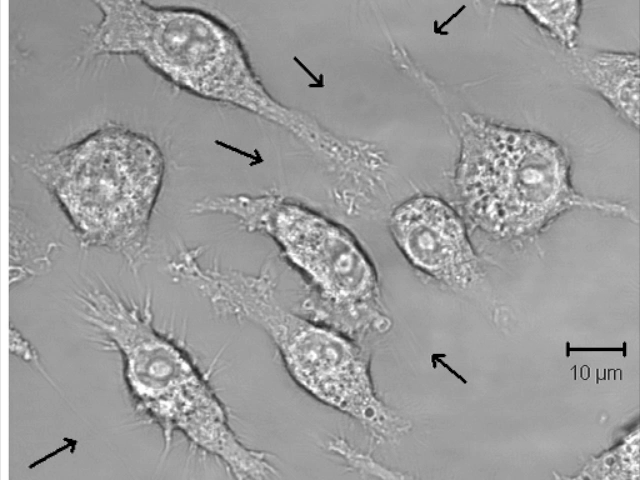

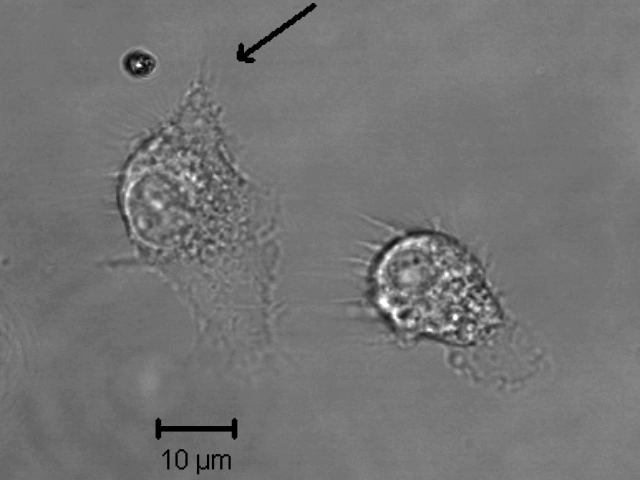
**

**Movie 3:** Murine macrophages cultured on a more rigid (76.8kPa) substrate. Movie demonstrate capture of a two beads via membrane projection and subsequent phagocytosis.

**Vil. Microarray Analysis** **Results**

Microarray analysis was performed of RAW 267.4 macrophages cultured on a 1.2kPa (less rigid) or 150kPa (more rigid) surface for 2 hours, 6 hours, or 18 hours (after removal from a plastic tissue culture flask). Three independent experiments (denoted a,b,c) were run for each condition, and mRNA was extracted and analyzed via Affymetrix GeneChip. For each microarray probe, we computed the linear correlation between the replicate profiles across all 6 (2 stiffnesses x 3 timepoints) conditions. For each Entrez GeneID that has >1 microarray probe representative, we selected the probe with the maximum linear correlation between replicate profiles to be its unique representative. Principle Component (PC) Analysis revealed that PC1 accounted for 34.8% of the variance, PC2 for 16.8% of the variance, and PC3 for 9.9% of the variance. The specific time points of culture were well distinguished along PC1 (see Figure 5A, and Figure 5B). Along PC2 the 2 hour and 18 hour time points showed similar levels of variance, while the 6 hour time point was distinguished between these two (Figure 5A) suggesting there were specific changes in gene activation at 6 hours culture time. Along PC3, one could distinguish cells cultured on the two rigidities, and importantly this variance increased over time and maximized at 18 hours (Figure 5B). This shows that substrate rigidity imparts a time-dependent influence on macrophage gene expression.

Geometric fold method analysis, using a cutfoff of 1.5 fold change revealed a list of 58 genes that were differentially regulated on a stiff (150kPa) versus soft (1.2kPa) substrate at 18 hours. 24 genes were up regulated on the stiff versus soft surface, and 34 genes were down regulated on the stiff versus soft surface. (see Table S1). Functional annotation clustering analysis using the NIH DAVID bioinformatics database[8], significant enrichment of this list for the ontological terms “response to wounding”, (P=0.0023, modified Fisher’s Exact P value) and “immune response”, (P=0.0085). There was no significant enrichment for phagocytosis or inflammation-related ontological terms.

In summary, these microarray data demonstrate that gene expression in macrophages changes upon adherence to a substrate according substrate rigidity. However, changes in gene expression do not readily predict any effects of substrate rigidity on phagocytosis or LPS response.

**Table S1**

| Gene | Fold Change 150kPa/1.2kPa | Gene | Fold Change 150kPa/1.2kPa |
| --- | --- | --- | --- |
| 2810403A07Rik | 1.50 | Mmp19 | -1.85 |
| Aldh2 | 1.51 | Olr1 | -1.91 |
| A130012E19Rik | 1.53 | F10 | -1.95 |
| 4432414F05Rik | 1.53 | Procr | -1.99 |
| LOC667118 | 1.54 | Clec4n | -2.01 |
| Eno2 | 1.54 | Ccr1 | -2.01 |
| 5830427D03Rik | 1.56 | Ifi202b | -2.11 |
| 6720401G13Rik | 1.56 | Rgs1 | -2.17 |
| Sfrs8 | 1.61 | Atp6v0d2 | -2.21 |
| D8Ertd575e | 1.61 | Egr2 | -2.40 |
| 2610319H10Rik | 1.63 | Serpinb9b | -2.45 |
| D3Ertd34e | 1.64 |  |  |
| Cit | 1.65 |  |  |
| D9Ertd306e | 1.67 |  |  |
| Nisch | 1.71 |  |  |
| Trim41 | 1.72 |  |  |
| Mecr | 1.72 |  |  |
| Slc5a3 | 1.77 |  |  |
| 9630010G10Rik | 1.80 |  |  |
| D330040H18Rik | 1.81 |  |  |
| A430108E01Rik | 1.81 |  |  |
| 4632427E13Rik | 1.96 |  |  |
| Hnrnpa1 | 2.21 |  |  |
| Hist1h3d | 2.34 |  |  |
| Bcar3 | -1.50 |  |  |
| Emp1 | -1.50 |  |  |
| Adh7 | -1.53 |  |  |
| Atf3 | -1.54 |  |  |
| Insl6 | -1.54 |  |  |
| Gpnmb | -1.55 |  |  |
| Rgs2 | -1.55 |  |  |
| Ccl2 | -1.56 |  |  |
| Cd83 | -1.56 |  |  |
| Lims2 | -1.58 |  |  |
| Ptgir | -1.58 |  |  |
| Layn | -1.58 |  |  |
| Angptl2 | -1.58 |  |  |
| Anxa4 | -1.59 |  |  |
| Hist1h2bc | -1.59 |  |  |
| Il1rn | -1.60 |  |  |
| Plk2 | -1.61 |  |  |
| Slc7a11 | -1.63 |  |  |
| Serpinb9 | -1.63 |  |  |
| Slc40a1 | -1.74 |  |  |
| Serpinb1b | -1.75 |  |  |
| Ccl7 | -1.81 |  |  |
| Irg1 | -1.83 |  |  |

**Supplemental Methods:**

**Reactive oxygen species assay.** RAW264.7 Cells were plated 100,000 per well of a 96 well plate and after overnight culture, 100μL of 10uM 2',7'-dichlorodihydrofluorescein diacetate (Molecular Probes (now Invitrogen, Carlsbad, CA)) working solution diluted in HBSS. Each well was ten loaded with IgG coated latex beads at an MOI of (20:1) and to the control well HBSS alone was added. Respiratory burst was measured using excitation wavelength of 490nm and emission at 525nm for 0, 20, 40, 80, and 120 minutes.

**Supplemental References:**

1. Lavnikova N, Prokhorova S, Helyar L, Laskin DL (1993) Isolation and partial characterization of subpopulations of alveolar macrophages, granulocytes, and highly enriched interstitial macrophages from rat lung. Am J Respir Cell Mol Biol 8: 384-392.

2. Cohen AB, Cline MJ (1971) The human alveolar macrophage: isolation, cultivation in vitro, and studies of morphologic and functional characteristics. J Clin Invest 50: 1390-1398.

3. Green GM, Kass EH (1964) The Role of the Alveolar Macrophage in the Clearance of Bacteria from the Lung. J Exp Med 119: 167-176.

4. Lambrecht BN (2006) Alveolar macrophage in the driver's seat. Immunity 24: 366-368.

5. Flannagan RS, Harrison RE, Yip CM, Jaqaman K, Grinstein S (2010) Dynamic macrophage "probing" is required for the efficient capture of phagocytic targets. J Cell Biol 191: 1205-1218.

6. Kress H, Stelzer EH, Holzer D, Buss F, Griffiths G, et al. (2007) Filopodia act as phagocytic tentacles and pull with discrete steps and a load-dependent velocity. Proc Natl Acad Sci U S A 104: 11633-11638.

7. Vonna L, Wiedemann A, Aepfelbacher M, Sackmann E (2007) Micromechanics of filopodia mediated capture of pathogens by macrophages. Eur Biophys J 36: 145-151.

8. Huang da W, Sherman BT, Lempicki RA (2009) Systematic and integrative analysis of large gene lists using DAVID bioinformatics resources. Nat Protoc 4: 44-57.
